# Robotic Heineke–Mikulicz stricturoplasty for refractory incisura angularis stenosis after sleeve gastrectomy: an anatomy-preserving case report

**DOI:** 10.1093/jscr/rjag728

**Published:** 2026-08-21

**Authors:** Carolina Baz, Angel Flores Huidobro, Alejandro Gandsas

**Affiliations:** Department of Surgery, Luminis Health Anne Arundel Medical Center, 2001 Medical Pkwy, Annapolis, MD 21401, United States; Department of Surgery, Luminis Health Anne Arundel Medical Center, 2001 Medical Pkwy, Annapolis, MD 21401, United States; Department of Surgery, Luminis Health Anne Arundel Medical Center, 2001 Medical Pkwy, Annapolis, MD 21401, United States

**Keywords:** sleeve gastrectomy, gastric stenosis, Heineke–Mikulicz stricturoplasty, gastric bypass

## Abstract

Gastric sleeve stenosis is an uncommon yet serious complication following sleeve gastrectomy (SG). Initial management typically involves endoscopic balloon dilation; however, refractory cases may require revisional surgery, often conversion to a Roux-en-Y gastric bypass (RYGB). We present the case of a 44-year-old woman with a symptomatic stenosis at the incisura angularis, 1 month after SG, refractory to three endoscopic balloon dilations. Given the localized nature of the stenosis and the patient’s preference to avoid bypass, she underwent robotic Heineke–Mikulicz stricturoplasty. The patient had an uncomplicated postoperative course and remained asymptomatic at follow-up. This case highlights the feasibility and advantages of stricturoplasty as an organ-preserving alternative to gastric bypass in selected patients with localized refractory sleeve stenosis, preserving gastrointestinal continuity and possibly reducing long-term nutritional considerations associated with bypass procedures. Further studies with longer follow-up are needed to better define patient selection, durability, and comparative outcomes with conversion to RYGB.

## Introduction

Sleeve gastrectomy (SG) was described in the 2000s and has become the most commonly performed bariatric operation worldwide due to its effectiveness, reproducibility, and relative technical simplicity [1, 2]. Despite these advantages, SG can present complications, including bleeding, leaks, and gastric stenosis, with an overall complication rate reaching up to 13% [3]. Among these, gastric stenosis is uncommon, occurring in ~0.7%–4% of patients, but can cause significant morbidity and substantially impair quality of life [3, 4].

Most gastric stenosis occur at the incisura angularis, where the sleeve is narrowest and most prone to angulation or functional obstruction. Proposed contributing factors include narrow tubularization, use of smaller bougies, staple line imbrication, sharp stapler angulation, twisting, edema, hematoma, ischemia, and scar formation during healing [1, 5]. Patients may present early or late after surgery with progressive dysphagia, reflux, nausea, vomiting, food or saliva intolerance, or excessive weight loss [6]. Although upper gastrointestinal contrast studies and computed tomography with oral contrast can aid diagnosis, upper endoscopy remains the diagnostic gold standard [1, 6].

Endoscopic balloon dilation is generally considered first-line therapy and is successful in many cases, although repeated sessions may be required [1, 7]. In refractory cases, revisional surgery may be necessary. Conversion to an Roux-en-Y gastric bypass (RYGB) has traditionally been the most frequently described surgical option, but anatomy-preserving alternatives, such as seromyotomy and stricturoplasty, have also been reported in selected cases [5, 8, 9].

We present a case of refractory incisura angularis stenosis following laparoscopic SG, managed with a robotic Heineke–Mikulicz stricturoplasty. This case illustrates the feasibility of a robotic anatomy-preserving approach as an alternative to conversion to gastric bypass in a selected patient.

## Case report

**Figure 1:**
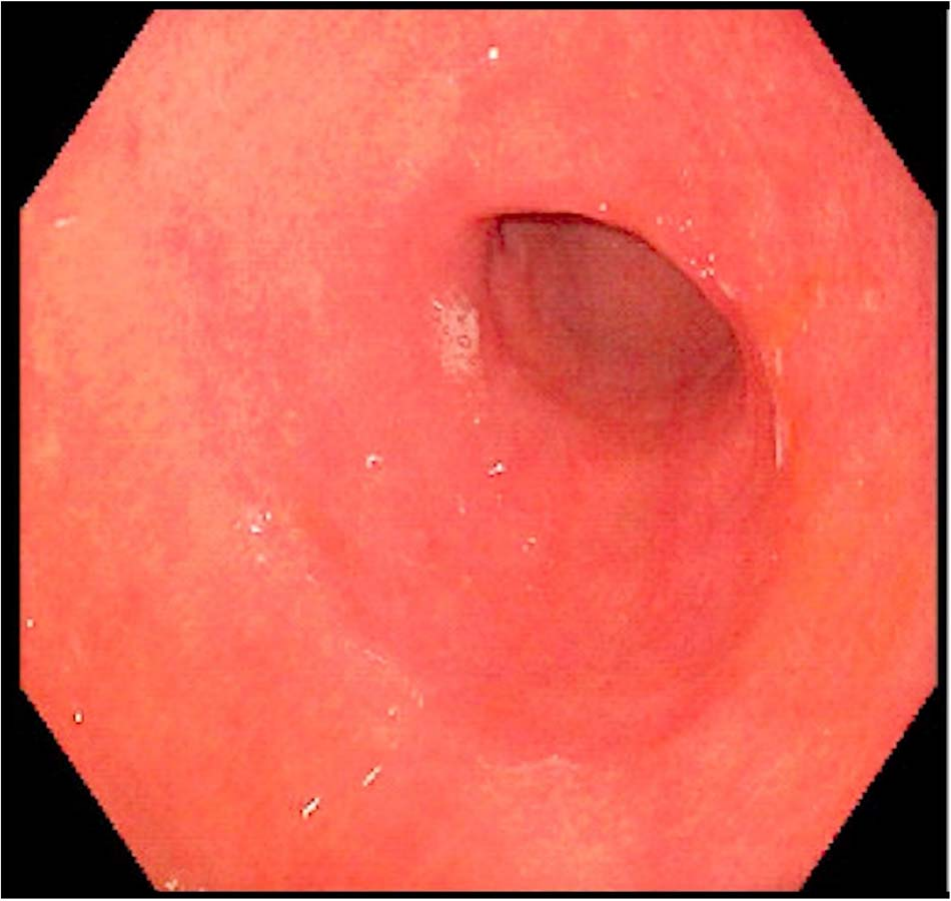
Preoperative endoscopic evaluation demonstrating functional narrowing at the incisura angularis after sleeve gastrectomy.

A 44-year-old woman with a body mass index of 37 kg/m^2^ and obesity-related comorbidities, including hypertension, hyperlipidemia, and lupus, underwent SG. One month postoperatively, she developed severe gastroesophageal reflux and progressive dysphagia. Upper endoscopy demonstrated a functional stricture at the incisura angularis (Fig. 1). She underwent three endoscopic balloon dilation attempts to 20 mm, each without durable symptomatic improvement, and was subsequently scheduled for robotic Heineke–Mikulicz stricturoplasty.

**Figure 2:**
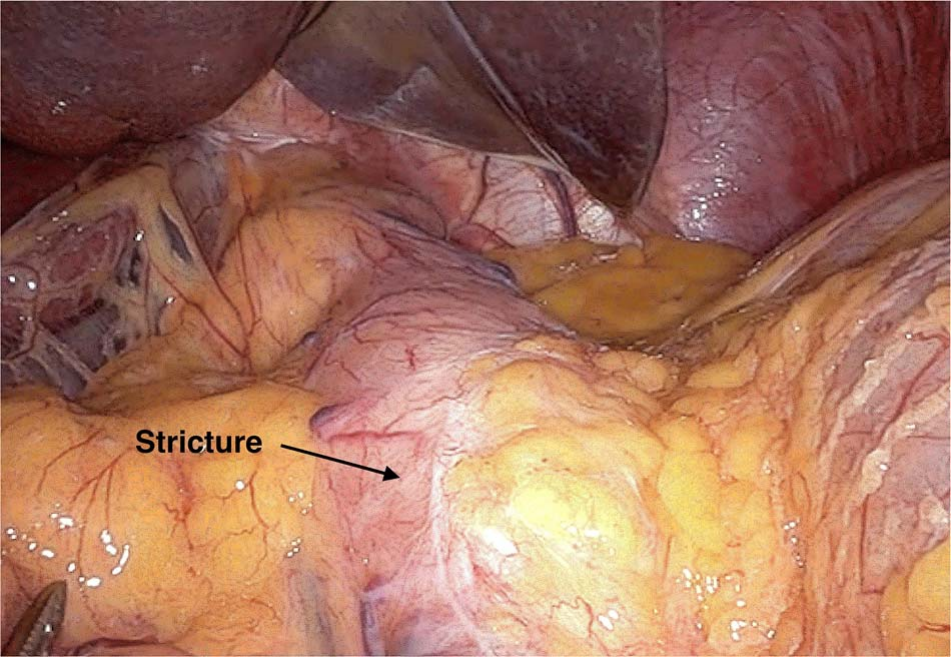
Intraoperative findings suggesting tethering or cinching of the sleeve at the incisura, potentially related to prior omentopexy.

A review of the index operation suggested that the sleeve had been appropriately constructed; however, the omentopexy appeared to have narrowed the incisura through a cinching effect, resulting in functional obstruction (Fig. 2). During the revisional procedure, the jejunum was clamped distal to the ligament of Treitz, and intraoperative endoscopy was performed to confirm the location of the stricture. The stenotic segment was marked ~3 cm proximal and distal to the area of narrowing. The robot was then docked to address the sleeve stricture.

**Figure 3:**
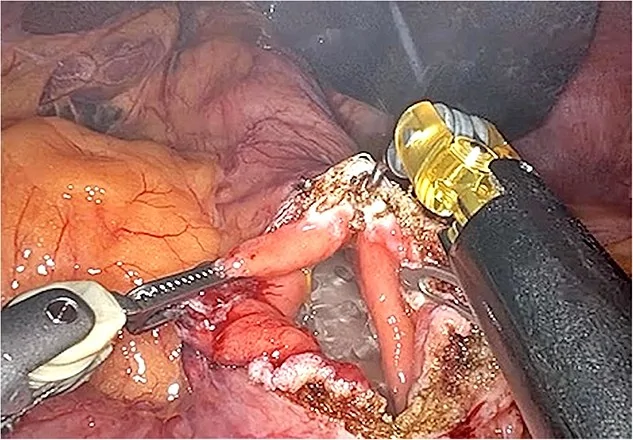
Longitudinal gastrotomy across the stenotic segment extending proximally and distally into healthy gastric tissue.

**Figure 4:**
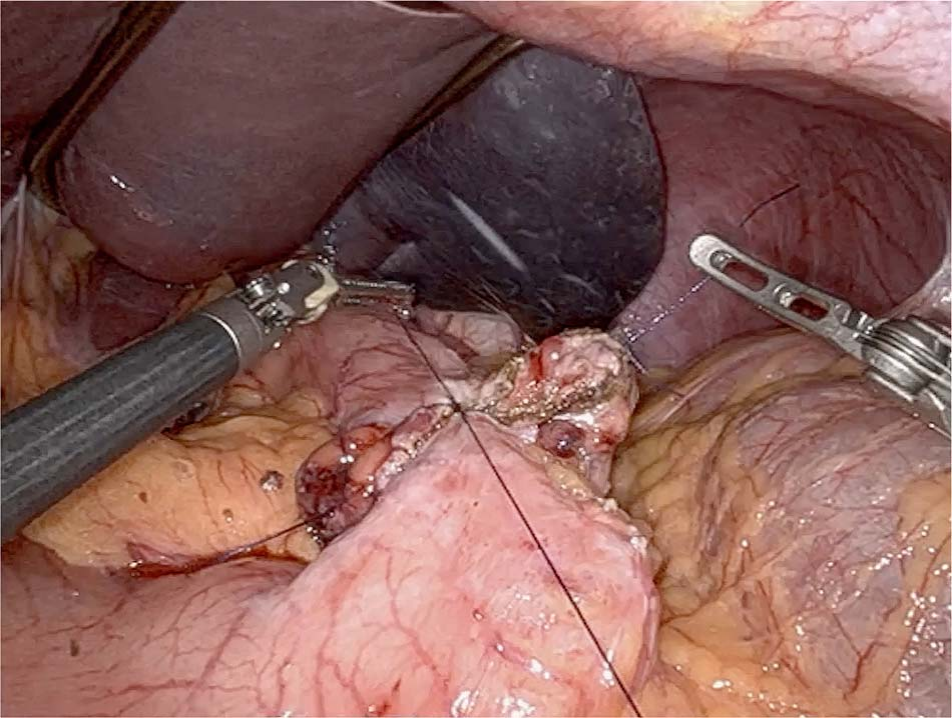
Transverse closure of the gastrotomy completing the Heineke–Mikulicz stricturoplasty.

A longitudinal gastrotomy was created across the stenotic segment, extending proximally and distally into healthy gastric tissue. A Heineke–Mikulicz-type stricturoplasty was performed by closing the longitudinal gastrotomy transversely (Fig. 3). Medial and lateral stay sutures were placed to provide traction and exposure, followed by a third stay suture to approximate the proximal and distal flaps and facilitate alignment. The transverse closure was completed with running 3-0 absorbable barbed sutures from both the lateral and medial aspects (Fig. 4). Intraoperative endoscopic insufflation under saline demonstrated no evidence of leak and confirmed adequate luminal patency.

**Figure 5:**
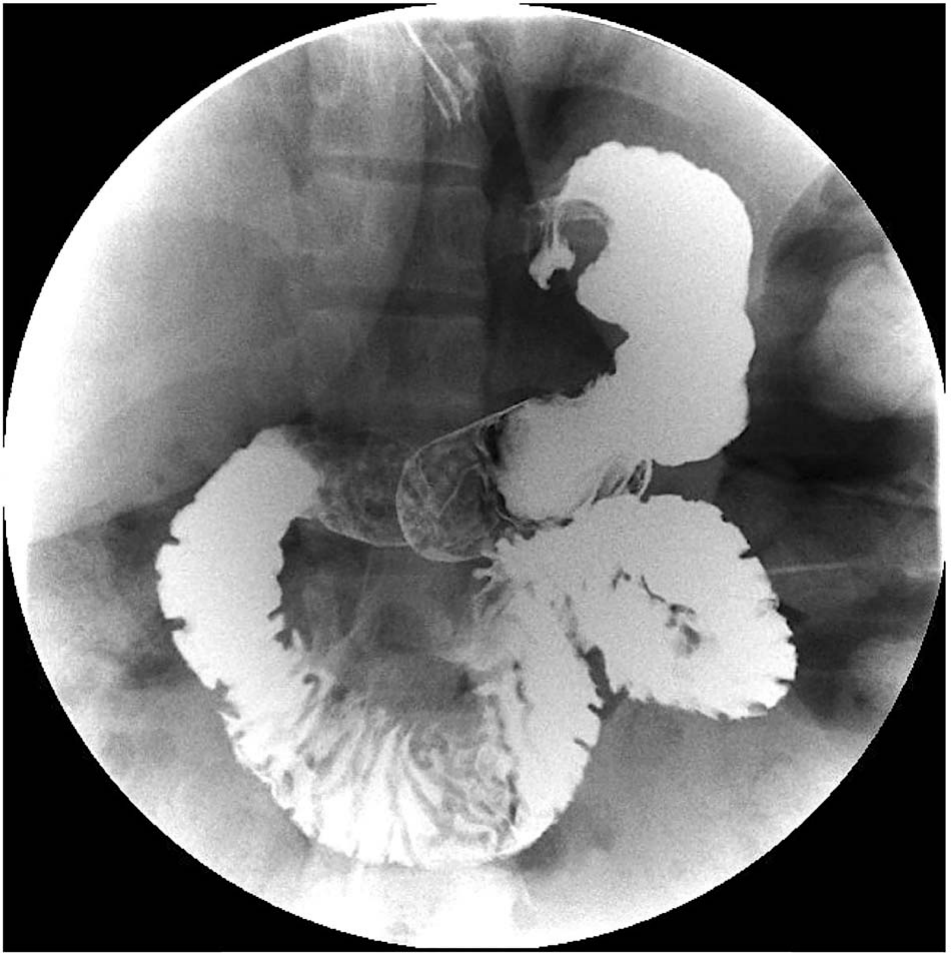
Postoperative upper gastrointestinal contrast study following robotic Heineke–Mikulicz stricturoplasty, demonstrating patent passage of contrast through the sleeve and into the duodenum without evidence of leak or obstruction.

A postoperative upper gastrointestinal contrast study demonstrated unobstructed passage of contrast through the repaired sleeve and into the duodenum, without evidence of leak or residual obstruction (Fig. 5). The patient had an uncomplicated postoperative course and was discharged on postoperative day 2 while tolerating a liquid diet. At 1-month follow-up, she was tolerating oral intake and denied dysphagia, gastroesophageal reflux, or vomiting.

## Discussion

Gastric stenosis following SG is considered one of the most serious complications after SG, along with staple-line leaks [6]. Although uncommon, sleeve stenosis can lead to substantial morbidity, and impaired quality of life [3, 4]. Strictures commonly occur at the incisura angularis, which is particularly vulnerable because it represents the narrowest portion of the sleeve and is prone to angulation, twisting, oversewing, or excessive tissue resection [9]. Consistent with this anatomic predisposition, Chang *et al.* [10] reported that 88.8% of stenotic sites in their series were located at the incisura angularis. Endoscopic balloon dilation remains the first-line treatment for sleeve stenosis; however, multiple sessions are often required, and a subset of patients ultimately fail nonoperative management [1, 7, 8].

Several technical measures may reduce the risk of sleeve stenosis. These include using an appropriately sized calibration bougie, avoiding excessive narrowing at the incisura angularis, maintaining a straight staple-line trajectory, and ensuring that the anterior and posterior gastric walls are symmetrically aligned to prevent sleeve rotation or spiraling. Staple-line reinforcement or imbrication should not constrict the lumen. When omentopexy is performed, sutures should be placed without excessive tension and should stabilize the sleeve without creating focal tethering, angulation, or distortion of its longitudinal axis. Intraoperative endoscopy or calibration-tube passage may also help identify narrowing or torsion before the operation is completed [8, 10, 11].

For refractory sleeve stenosis, conversion to RYGB has traditionally been the most frequently described revisional operation [6, 8]. While effective, it significantly alters gastrointestinal anatomy and exposes patients to additional operative complexity and long-term nutritional implications, including malabsorption [8]. Therefore, in selected patients, anatomy-preserving alternatives may be desirable [8, 12]. Among these, seromyotomy and stricturoplasty have been reported as feasible approaches. Dapri *et al.* [12] and Vilallonga *et al.* [8] have shown encouraging outcomes with seromyotomy in patients with long stenosis after SG. Conversely, Sudan *et al.* [5] described robotic Heineke–Mikulicz stricturoplasty as an alternative method for managing SG stenosis, favoring the robotic approach for its advantages in precise suturing and 3-dimensional visualization. Recent publications continue to emphasize that these alternative surgical interventions may effectively resolve obstructive symptoms while avoiding conversion to gastric bypass [9].

In our case, review of the index operation suggested that the omentopexy may have contributed to functional obstruction. We elected to perform a Heineke–Mikulicz stricturoplasty because the stenosis was localized and amenable to transverse widening. This technique allowed direct enlargement of the narrowed lumen while preserving the physiologic continuity of the gastrointestinal tract. The robotic platform provided several advantages in this revisional setting, which is usually complicated by adhesions, limited working space, and distorted anatomy. Its enhanced visualization, wristed instrumentation, and improved ergonomics facilitated adhesiolysis and precise intracorporeal suturing [5, 13]. These advantages may make robotic stricturoplasty a valuable alternative for carefully selected patients with functional sleeve obstruction caused by angulation, torsion, or external fixation.

This case also highlights the importance of technical considerations during omentopexy. Although omentopexy may be used to stabilize the sleeve and reduce twisting, excessive tension, wide suture spacing, or fixation that alters the sleeve axis may contribute to functional obstruction. Recognizing the mechanism of obstruction allowed a targeted repair.

The findings from this case should be interpreted cautiously, as this is a single-patient experience. In addition, because a standardized preoperative upper gastrointestinal contrast study was not obtained, a direct radiographic comparison before and after stricturoplasty was not possible. Nevertheless, postoperative contrast imaging demonstrated unobstructed passage through the repaired sleeve without evidence of leak or residual obstruction.

We believe that robotic stricturoplasty may represent a useful, anatomy-preserving option for selected patients with localized, refractory sleeve stenosis, particularly when the obstruction appears to be related to angulation, torsion, or external fixation rather than diffuse or long-segment narrowing.

This research received no specific grant from funding agencies in the public, commercial, or not-for-profit sectors.
